# Itaconate Suppresses Formation of Neutrophil Extracellular Traps (NETs): Involvement of Hypoxia-Inducible Factor 1α (Hif-1α) and Heme Oxygenase (HO-1)

**DOI:** 10.3389/fimmu.2022.864638

**Published:** 2022-06-28

**Authors:** Gabriela Burczyk, Iwona Cichon, Elzbieta Kolaczkowska

**Affiliations:** Laboratory of Experimental Hematology, Institute of Zoology and Biomedical Research, Jagiellonian University, Krakow, Poland

**Keywords:** itaconic acid, neutrophils, heme oxygenase, HO-1, hypoxia-inducible factor, Hif-1α, nuclear factor erythroid 2-related factor 2, Nrf2

## Abstract

Neutrophil extracellular traps (NETs) immobilize pathogens during early stages of systemic inflammation but as the reaction progresses they become detrimental to endothelial cells and the organ-specific cells. For this reason it would be of importance to control their formation by either physiological or pharmacological means. Endogenously, formation of NETs is under control of cellular and whole organism metabolism as shown previously in the course of bacterial systemic inflammation, obesity or the combination of the two. Numerous leukocytes are subjected to immunometabolic regulation and in macrophages exposure to lipopolysaccharide (LPS) leads to two breaks in the Krebs cycle that impact this cell functioning. As a consequence of the first break, anti-microbial itaconic acid (itaconate) is produced whereas the second break activates hypoxia-inducible factor-1α (Hif-1α). In turn, itaconate activates transcription of the anti-inflammatory nuclear factor erythroid 2-related factor 2 (Nrf2) which upregulates cyto-protective heme oxygenase (HO-1). Here we report that exogenously added derivative of the itaconic acid, 4-octyl itaconate (4-OI), diminishes formation of NETs by neutrophils of either normal (lean) or obese mice, and independently of the age of the animals or immunoaging. Elucidating the mechanism of this inhibition we unravel that although Nrf2/HO-1 expression itself is not altered by 4-OI, it is up-regulated when compared against the NET formation while Hif-1α is downregulated in 4-OI-pre-treated LPS-stimulated neutrophils in either way. We further show that blockage of Hif-1α by its specific inhibitor diminishes NET release as does inhibition by 4-OI. Also inhibition of HO-1 activity correlates with diminished LPS-induced NET release upon pre-treatment with 4-OI albeit LPS alone induced NETs are not HO-1-dependent. In summary, we unravel that 4-OI inhibits NET formation by murine neutrophils independently of their origin (health vs. metabolically challenged animals) and the age of individuals/immunosenescence *via* inhibition of Hif-1α and induction of HO-1.

## Introduction

Immunometabolism concerns the multi-level interactions between the immune system and metabolic processes, with an emphasis on actions related to the functioning of leukocytes (1). Research on immunometabolism is considered in terms of the impact of leukocytes own metabolism on their effector functions (2) and the influence of leukocytes on metabolic processes within themselves and the body (3). Disturbance of the immune-metabolic response can lead to either numerous pathological conditions or accompany them. Exemplary conditions of altered immunometabolism are obesity (metabolism → immunity) and sepsis/systemic inflammation (immunity → metabolism). Sepsis is defined as “life‐threatening organ dysfunction caused by a dysregulated host response to infection” (4), and in humans it carries a high mortality risk of up to 40% (5). Despite the fact that many pathophysiological changes in both experimental and clinical sepsis have been described, their detailed mechanisms are still obscure, making it difficult to develop specific (“targeted”) treatment strategies (6).

The studies conducted so far provide evidence that deaths due to sepsis are not caused by microorganisms themselves, but due to continuous activation of the innate immune response which results in robust inflammation leading to damage to the host’s cells and organs (7). Importantly, numerous evidence points to neutrophil extracellular traps (NETs) released by neutrophils as important pathogenic factors in the course of sepsis (8). The backbone of NETs is composed of extracellular DNA to which proteins are attached, including nuclear histones and proteins (e.g. cathepsin G, neutrophil elastase, NE) released from the neutrophil granules (9). Initially NETs were believed to be beneficial through their ability to trap and immobilize pathogens, but later studies have shown that they can also contribute to organ and tissue damage (7). In line with this, intravital microscopy imaging (IVM) revealed that NETs released by highly activated neutrophils during sepsis contribute to damage to the vascular endothelium of the liver as well as hepatocytes *via* NET attached NE (7) and histones (10). Thus, the ability to control this process, pharmacologically or metabolically, is an important subject of studies as well as an indirect goal of this research.

Another important condition accompanied by both immunometabolic regulation and altered release of NETs is obesity that is characterized by low-grade inflammation initiated in the adipose tissue that later spreads to other organs causing their damage (11). The process is initiated by the release of NE and infiltration of neutrophils that then recruit macrophages into the adipose tissue (12). We showed that during sepsis less neutrophils are engaged in the immune response in the liver, they display diminished interactions with platelets, and consequently release less NETs which (as we proposed) explains, at least partially, the “obesity paradox in sepsis” (13). However, isolated neutrophils of obese mice release similar, if not higher, quantities of NETs (13) suggesting that the above effect is not due to an intrinsic impairment.

In the context of studies on neutrophil metabolism, it is important to note that neutrophils have a very small number of mitochondria [5-6 versus 1000 in hepatocytes (14)] which suggests that they mainly relay on glycolysis for energy (15). In line with the above, NET formation and lactate accumulation during lipopolysaccharide (LPS)-induced sepsis was inhibited by sodium oxamate (an inhibitor of lactate dehydrogenase) underlining the importance of glycolysis in the formation of NETs (16). However, some studies suggest a possibility of other metabolic pathways being engaged to some extent, such as the pentose phosphate pathway (PPP), the Krebs cycle, oxidative phosphorylation (OXYPHOS), and fatty acid oxidation (FAO) (2). The experiments conducted by our group revealed that obesity predisposes neutrophils isolated from septic mice to spontaneously release NETs without any stimulation. And that indeed, this process is controlled not only by glycolysis but also by PPP pathway and FAO. Moreover a cross-talk between various metabolic pathways also might impact NET release independently of obesity (17). In this context it is of importance that glucose metabolism by PPP involves heme oxygenase (HO-1) (18) which is responsible for the degradation of heme released from senescent or damaged erythrocytes into less toxic free iron, bilirubin and carbon monoxide (19). Therefore HO-1 is considered an anti-inflammatory enzyme. Expression of HO-1, as of many other cyto-protective genes, is regulated by nuclear factor erythroid 2-related factor 2 (Nrf2) which up-regulates expression of genes coding for anti-oxidant, anti-inflammatory and detoxifying proteins (20).

In the current study we aimed at studying impact of immunometabolism on NET formation from yet another angle – can we metabolically impact neutrophils so the release of the traps is diminished? We preselected itaconic acid (itaconate, methylidenesuccinic acid) as a possible metabolic regulator since it was shown to immunosuppress macrophages, another important cellular players of the innate immunity. Itaconic acid is endogenously produced by macrophages during the “broken Krebs cycle” as an intermediate metabolite upon LPS treatment (21). The formation from citrate of the intermediate cis-aconitane (cis-aconitic acid), being a precursor for itaconic acid, is catalyzed by aconitase. Cis-aconitic acid, under the influence of the cis-aconitic acid decarboxylase (CAD) enzyme, undergoes decarboxylation and resulting itaconic acid is transported to the cytosol by the oxoglutarate carrier (OGC) where it acts as an activator of transcription factors such as activating transcription factor 3 (ATF3), finally inhibiting the release of pro-inflammatory cytokines (22). It was reported that itaconate has the ability to alkylate cysteine residues on multiple proteins, including aldolase A (ALDOA) (23), glyceraldehyde 3-phosphate dehydrogenase (GAPDH) (24), NLR family pyrin domain-containing protein 3 (NLRP3) inflammasome (25), and receptor-interacting serine/threonine-protein kinase 3 (RIPK3) (23). In the empirical studies, 4-octyl itaconate (4-OI), a derivative of the itaconic acid, is used. It was shown that 4-OI activates transcription of the anti-inflammatory factor - Nrf2 in human and mouse macrophages by modifying the cytoskeleton Kelch Like ECH Associated Protein 1 (KEAP1; physiologically present in the cytoplasm where it forms a complex with Nrf2). The cysteines present in the structure of the KEAP1 protein are alkylated by itaconic acid which leads to the accumulation of Nrf2 and its translocation to the cell nucleus (26). This process leads to the transcription of numerous cytoprotective genes such HO-1 thus stabilizing the redox potential (26). Activated Nrf2/HO-1 pathway leads to the inhibition of NFκB signaling (27) and may protect the cell from H_2_O_2_ cytotoxicity (20). Moreover, hypoxia-inducible factor-1α (Hif-1α) can be regulated by Nrf2 (28) whereas HO-1 can stabilize it (29). Hif-1α, is the master transcriptional regulator of cellular and developmental response to hypoxia which promotes tissue oxygenation and induces transcription of genes involved in cell proliferation and survival, as well as glucose metabolism (30).

Herein we report the impact of itaconate acid (4-OI) on neutrophil physiology, metabolism and basic immune functions with emphasis on NETs formation. Not only did we test them on neutrophils collected from healthy mice but also on the some cells collected from obese mice and their respective lean counterparts (age and sex-matched).

## Materials and Methods

### Mice

C57BL/6J male mice (three weeks old) were purchased from Charles River Laboratories (Sulzfeld, Germany; *via* AnimaLab). Animals were randomly divided into three groups and were fed either standard diet (Altromin, Lage, Germany, C1324, Fat 11%, Carbohydrates 65%, Protein 24% of kcal) for up to 8 weeks, control diet (=normal diet, lean mice; Altromin, C1000, Fat 13%, Carbohydrates 67%, Protein20% of kcal) or a high-fat diet (obese mice; Altromin, C1090—60, Fat 60%, Carbohydrates 24%, Protein 16% of kcal) for at least six months. The pelleted food and tap water were available *ad libitum*. Mice on a normal and high-fat diets were weighed every seven days to monitor the increase in the body mass in obese animals in comparison to lean mice (detailed data published in (13)). Animals were housed at a constant temperature (21-22°C) and under the conditions of a daily cycle of 12h light/12h darkness. All procedures for experimental animals were approved by the Local Ethical Committee No. II in Krakow (293/2017) and were in compliance with the EU Animal Care Guidelines.

### Bone Marrow Derived Neutrophil Isolation

The experiments were performed on neutrophils isolated from the bone marrow (BM) of healthy unstimulated mice. Prior to neutrophil isolation the animals were anesthetized by intraperitoneal (i.p.) injection of a mixture of ketamine hydrochloride (Biowet Pulawy, Pulawy, Poland) solution at a dose of 200 mg/kg body weight and xylazine hydrochloride (antiMEDICA, Südfeld, Germany) at a dose of 10 mg/kg body weight, and then cervical dislocation was performed. The lower limbs were separated from the rest of the body and after cleaning from skin and muscle tissue (to obtain tibia and femur bones) they were placed on ice in Petri dishes filled with HBSS (–) i.e. without Ca^2+^/Mg^2+^ (Lonza Bioscience, Basel, Switzerland). Subsequently, the ends of the bones were cut off and the bone marrow was flushed into a new dish containing ice-cold HBSS (–) using 25G needle. The BM was disintegrated with a 20G needle. The resulting cell suspension was transferred to falcon tubes and centrifuged at 4°C, 1300 rpm, 6 minutes. After centrifugation, the pellet was suspended in 5 ml of 0.2% NaCl (Chempur, Piekary Slaskie, Poland) for about 30 seconds to lyse the erythrocytes, then 5 ml of 1.6% NaCl was added to restore osmolarity and immediately centrifuged at 4°C, 1400 rpm, 7 minutes. The obtained cell pellet was suspended in 2 ml of 52% Percoll (GE Healthcare, Chicago, IL, USA). Then, 2 ml of Percoll solutions at 69% and 78% concentrations were applied successively to the previously prepared suspension of bone marrow cells in 52% Percoll, with a glass Pasteur pipette, followed by centrifugation for 30 minutes at 4°C, 2600 rpm. In the next step, with a Pasteur pipette, the fraction of cells localized between 69% and 78% Percoll layers was collected into new tubes and replenished with HBSS (-) to ¾ volume and centrifuged again for 6 minutes at 4°C, 1500 rpm. The pellets of the obtained neutrophil population were resuspended in 1 ml of HBSS (+) i.e. with Ca^2+^/Mg^2+^. Counts, viability and purity of the isolated cells were determined using the hemocytometric method (Bürker chamber) by staining, respectively, with Türk’s solution (0.1% crystal violet (Sigma–Aldrich, Saint Louis, MO, USA) in 3% acetic acid) and 0.4% Trypan blue solution, used in a 1:1 ratio (Sigma–Aldrich). The neutrophil viability was ~ 99% (Trypan blue) and purity of isolated neutrophils was 98-99% (Türk/hemocytometer) in each experiment. There were no differences in neutrophil purity between young and old mice (**Supplementary Figure 1**).

The experiments were performed in 96-well plates. In some experiments, coverglasses (5 mm in diameter) were placed at the bottom of the wells prior to cell seeding at a density of 50,000 cells/well. Before stimulation, the plates were placed in an incubator for 30 minutes under standard conditions (37°C, 5% CO_2_) to adhere.

### Treatment With 4-Octyl Itaconate

Prior to LPS stimulation bone marrow derived neutrophils were treated for 1h with various concentrations of 4-octyl itaconate (4-OI) - 62,6 µM and 125 µM (Cayman Chemical, An Arbor, MI, USA) reconstituted in dimethyl sulfoxide (DMSO, Sigma–Aldrich). The concentrations were chosen based on their efficacy in previous studies on murine macrophages (26). Further dilutions were preprepared in HBSS. The remaining control groups of cells were treated with dimethylsulfoxide (DMSO) solvent in the same volume as it was used in 4-OI working solution (62,5 µM; 125 µM).

### Inhibitors of Metabolic Pathways

In some experiments inhibitors of various pathways were applied in either of the following regimes (always with 1 hour incubation in between treatments): addition of an inhibitor prior to LPS (inhibitor→LPS), addition of an inhibitor after 4-OI, then followed by LPS (4-OI→inhibitor→LPS). Heme oxygenase 1 (HO-1) inhibitor - Tin Protoporphyrin IX dichloride (SnPP, 20 µM, Santa Cruz Biotechnology, Dallas, TX, USA) and Hypoxia inducible factor-1α (Hif-1α) inhibitor (CAS Number: 934593-90-5, 10 µM, Santa Cruz Biotechnology) were used upon reconstitution in DMSO. Further dilutions were preprepared in HBSS. The remaining control groups of cells were treated with dimethylsulfoxide (DMSO) solvent in the same volume as it was used in 4-OI working solution (62,5 µM; 125 µM). The effectiveness of the Hif-1α inhibitor was tested by staining the LPS stimulated cells for Hif-1α expression with immunocytochemistry as described below. The inhibitor did significantly inhibit expression of Hif-1α (**Supplementary Figure 2**).

### LPS Stimulation

After incubation with the metabolic pathway regulators, neutrophils were stimulated for 6 hours with lipopolysaccharide (LPS, *Escherichia coli serotype* 0111:B4; Sigma–Aldrich) at a final well concentration of 75 µg/ml.

### Neutrophil Adhesion: Crystal Violet Assay

The neutrophil adhesion was verified with the Crystal violet assay (CV, Avantor, Gliwice, Poland). After 6-h incubation with 4-OI supernatant was removed from the cells and they were fixed by adding 50 μl of 100% methyl alcohol (KrakChemia, Kraków, Poland) to each well for 10 min. Methanol was removed and the adherent cells were stained by a 5-minute incubation with CV solution (25 mg CV in 5 ml 20% methyl alcohol). At the end of incubation, the plate was gently washed in running tap water and air-dried. On the next day, 100 μl of 100% methanol was added to each well to extract the crystal violet from the inside of the cells. The optical density (O.D.) was measured in a spectrofluorometer Infinite 200 Pro (Tecan, Austria, Gmbh) at 570 nm.

### Neutrophil Viability: PrestoBlue^®^ Assay

The viability of cells was measured with PrestoBlue^®^ assay (Invitrogen, Waltham, MA, USA). After 5.5 h of incubation with 4-OI 10 µl (1:10 ratio) of PrestoBlue^®^ was added to each well; the plate was placed in an incubator (5% CO_2_, 37°C) for 0.5 h. Next, fluorescence was measured at 540/610 nm in a spectrofluorometer Infinite 200 Pro, 6 hours after the addition of the LPS. Data is expressed as fluorescence arbitrary units (AU).

### Neutrophil Metabolic Activity: MTT Assay

The metabolic activity of cells was studied with the MTT assay (Sigma–Aldrich). After 3 h of incubation with 4-OI 20 μl of MTT solution at a concentration of 5 mg/ml was added to each well. Then the plate was placed in an incubator for three hours, after which the supernatant was removed and the resulting formazan crystals were thoroughly dissolved in 100 μl 0.04 M HCl (Avantor) in isopropanol (Chempur). The optical density (O.D.) was measured in a spectrofluorometer Infinite 200 Pro at 570 nm.

### Neutrophil Capacity to Perform Oxidative Burst: NBT Assay

The neutrophil capacity to perform oxidative burst was verified with the NBT assay (Sigma–Aldrich). After 5 h incubation with 4-OI 10 µl of NBT at a concentration of 10 mg/ml was added to each well. Then the plate was placed in an incubator for one hour. After incubation supernatant was removed and 100 µl of 100% methanol was added to each well for 15 min to fix the cells. Methanol was removed and wells were rinsed twice with 70% methyl alcohol after which the plate was allowed to air dry. In the last step, the formed formazan crystals were dissolved by adding 120 µl of 2M KOH (Chempur) and 140 µl of DMSO to each of the wells. The optical density (O.D.) was measured in a spectrofluorometer Infinite 200 Pro at 590 nm.

### Immunocytochemical Staining of NETs

Immediately after 6 hours of incubation, the cells were fixed in a sequence of 1% (2 min), 2% (10), and 3% (20 min) paraformaldehyde (PFA, Alfa Aesar, Haverhill, MA, USA), and washed in PBS (Gibco, Waltham, MA, USA). Subsequently extracellular staining for citrullinated histones H3 (citH3) and extracellular DNA (extDNA) was performed. The coverglasses were washed twice with PBS and incubated in 3% BSA/PBS (BSA, bovine serum albumin; Sigma–Aldrich) for 45 minutes in a humid chamber at room temperature. Next, a drop of rabbit polyclonal anti-histone H3 (citrulline R2+R8+R17) antibody (Abcam, Cambridge, UK) diluted 1:200 in 1% BSA/PBS was added on the fixed cells. This was followed by an 18-hour incubation with primary antibodies in a humid chamber (at 4°C). Subsequently, the coverglasses were washed twice in PBS (5 min), and then soaked with secondary antibodies prepared in 1% BSA/PBS (1:300) - Cy3-conjugated goat anti-rabbit IgG (H+L) antibodies (Jackson ImmunoResearch Laboratories, Baltimore Pike, OH, USA) for one hour at room temperature in the dark. As a last step, coverglasses were washed with PBS and incubated with Sytox green (5 µM, ThermoFisher Scientific, Waltham, MA, USA) to stain extracellular DNA. After five minutes, the coverglasses were washed for the last time in PBS, and after the procedure was completed, they were placed on drops of the VECTASHIELD Mounting Medium (Vector Laboratories, Burlingame, CA, USA).

### Immunocytochemical Staining of HO-1, Nrf2 and Hif-1α

Immunostaining of heme oxygenase (HO-1), nuclear factor erythroid 2-related factor 2 (Nrf2) and Hypoxia inducible factor-1α (Hif-1α) was performed. Neutrophils seeded on slides were permeabilized by bathing in TBS (0,025% Triton X-100, 3,5% Na_2_HPO_4_×12H_2_O, 0,42% Na_2_HPO_4_×1H_2_O, 0,025% BSA, 5% NaCl, dH_2_O) for 5 min and blocked by incubation with 3% BSA/PBS (45 min). Subsequently, overnight incubation with rabbit polyclonal HO-1 antibody (diluted 1:250 in 1% BSA/PBS; Enzo Life Sciences, Farmingdale, NY, USA), rabbit monoclonal Nrf2 antibody (diluted 1:200 in 1% BSA/PBS; Cell Signaling Technology, Danvers, MA, USA) or rabbit monoclonal Hif-1α antibody (diluted 1:400 in 1% BSA/PBS; Cell Signaling Technology) was performed (4°C, in a humid chamber). After 18 hours the slides were washed 2 times (5 min) in PBS and bathed in the secondary antibody goat anti-rabbit IgG (H+L) (Cy3) (diluted 1:300 in 1% BSA/PBS) for 1 h. Finally, coverglasses were washed with PBS, and then Sytox green (5 µM, 5 min) was added to stain for extDNA. As a last step, slides were washed in PBS and mounted with VECTASHIELD Medium. In order to verify specificity of the signal produced by the primary and secondary antibodies the experiments were run in which either Rabbit IgG Isotype Control (Invitrogen, Waltham, MA, USA) was used or the primary antibodies were omitted, respectively. In either case, no unspecific binding was detected (**Supplementary Figure 3**).

### Microscopic Analysis

After immunocytochemical staining, fluorescent signal was detected with a ZEISS Axio Examiner.Z1 upright microscope equipped with confocal spinning disk device DSD2 (with ZEISS EC Plan‐NEOFLUAR 20×/0.5 air objective) in two channels: GFP (green fluorescent protein; 482/18 nm Ex, 525/45 nm Em); RFP (red fluorescent protein; 561/54 nm Ex, 609/54 nm Em) using IQ Live Cell Imaging software (Andor, Oxford Instruments, Abingdon, UK). The photos obtained from immunocytochemical staining were analyzed using the ImageJ v1.53a program (National Institutes of Health, USA). The images were converted to grayscale (8-bit type) and the positive signal was estimated using the threshold function. In the case of HO-1, Nrf2 and Hif-1α immunostaining, tresholded, positive signals in images from GFP (extDNA) and RFP (HO-1/Nrf2/Hif-1α) channels were calculated using the ImageJ. The RFP/GFP ratio [%] was counted to determine the level of proteins expression in conversion to the amount of released NETs.

### Statistics

All data are presented as mean values ± SD. Data were compared by unpaired two-tailed Student’s t test (*p ≤ 0.05, **p ≤ 0.01, ***p ≤ 0.001, ****p ≤ 0.0001) and one way analysis of variance (ANOVA) with Bonferroni multiple comparisons *post hoc* test (ANOVA: any two means that do not share the same letter are significantly different). Statistical significance was set at p < 0.05.

## Results

### Itaconate Inhibits NET Formation

**Figure 1:**
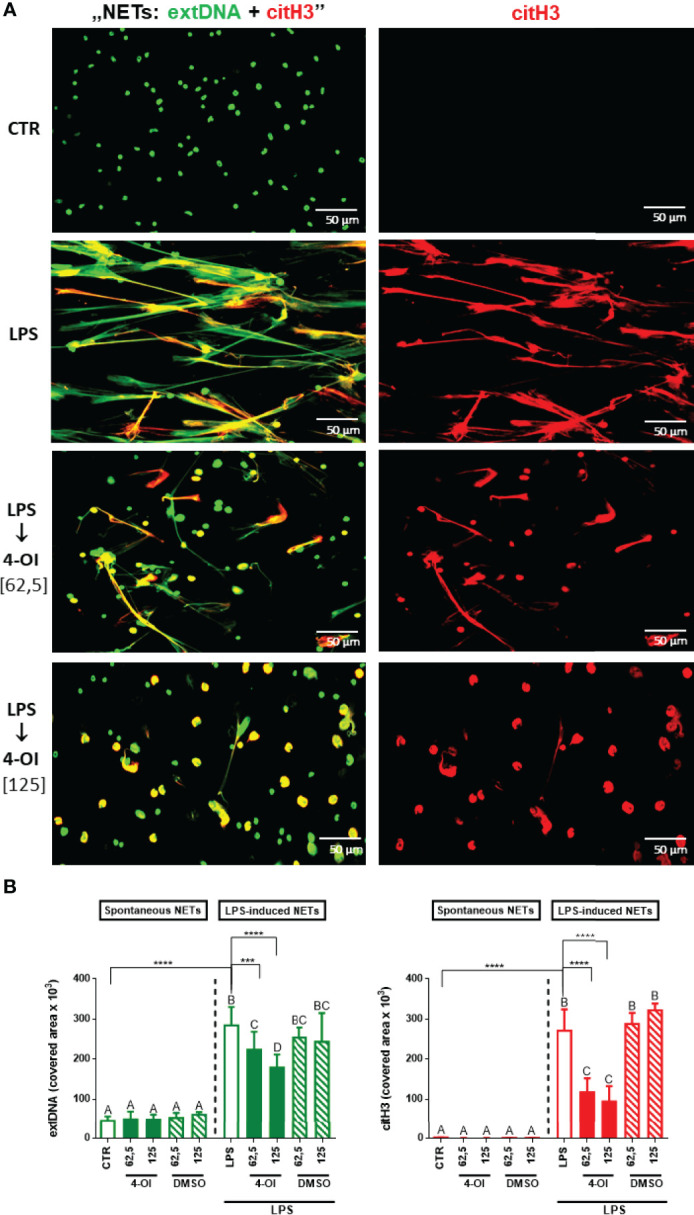
Effect of the Krebs cycle metabolite 4-OI itaconate (4-OI) on the ability to form NETs by neutrophils isolated from healthy mice on a standard diet. Neutrophils were either pre-treated with various concentrations of 4-OI (62,5 µM; 125 µM; for 1h), incubated with lipopolysaccharide (LPS) at a concentration of 75 μg/ml (for 6h), pre-treated with 4-OI and stimulated with LPS (4-OI→LPS) or left unstimulated (CTR). The remaining control groups of cells were treated with dimethylsulfoxide (DMSO) solvent in the same volume as it was used in 4-OI working solution (62,5 µM; 125 µM). Representative images of NETs formed by LPS in the presence 4-OI are shown in **(A)**. Extracellular DNA (extDNA) is shown in green while citrullinated histone H3 (citH3) in red. To visualize co-localization of NET components, the images from each channel were overlaid (NETs: extDNA + citH3). **(B)** Quantification of NET formation: area covered by the extDNA and citH3 signal. The results are expressed as the mean values ± SD; n≥3. Values significantly different between the groups (p < 0.05) according to one-way ANOVA (*post hoc* Bonferroni test) are designated by letters, where the same letter indicates no differences between groups (different letters indicate statistical differences). Additionally asterisks indicate significant differences between groups according to unpaired two-tailed Student’s t-test (***p ≤ 0.001, ****p ≤ 0.0001).

As expected, LPS treatment induced robust release of NETs by neutrophils, estimated by co-localization of extracellular DNA (extDNA) and citrullinated histones H3 (citH3) - whereas hardly any traps were observed in the vicinity of untreated cells (**Figures 1A, B**). When the cells were pre-treated with 4-octyl itaconate (4-OI) prior to LPS stimulation, NET formation was significantly inhibited and this was concentration-dependent. 4-OI alone did not induce NETs and neither DMSO itself impacted NET formation (**Figures 1A, B**). In the studies two concentrations of 4-OI were tested (62,5 µM and 125 µM) whereas when the concentration was increased to 300 and 500 µM this lead to neutrophil death (data not shown).

### Itaconate Hardly Impacts Neutrophil Numbers and Basal Metabolism

**Figure 2:**
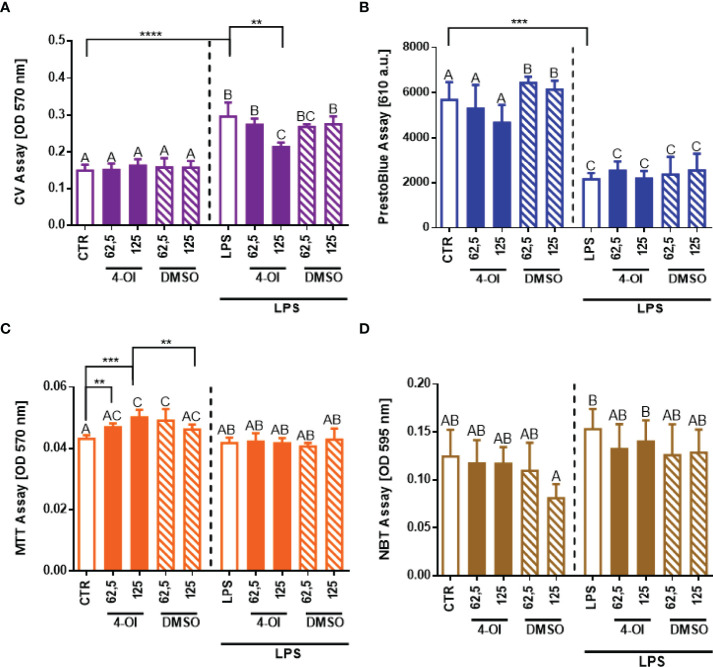
Effect of the Krebs cycle metabolite 4-octyl itaconate (4-OI) on the neutrophil adherence to the surface, viability, metabolic activity and reactive oxygen species (ROS) production by neutrophils. The analyses were performed with crystal violet assay (CV) **(A)**, PrestoBlue **(B)**, MTT **(C)** and NBT **(D)** assays respectively. Neutrophils isolated from the bone marrow of mice on a standard diet were treated with 4-OI at concentrations of 62,5 µM and 125 µM for 1 hour. The remaining control groups of cells were treated with dimethylsulfoxide (DMSO) solvent in the same volume as it was used in 4-OI working solution (62,5 µM; 125 µM) or they were left unstimulated (CTR). Some of the cells were subsequently stimulated for 6 hours with lipopolysaccharide (LPS) at a concentration of 75 μg/ml. The results are expressed as the mean values ± SD; n≥3. Values significantly different between the groups (p < 0.05) according to one-way ANOVA (*post hoc* Bonferroni test) are designated by letters, where the same letter indicates no differences between groups (different letters indicate statistical differences). Additionally asterisks indicate significant differences between groups according to unpaired two-tailed Student’s t-test (**p ≤ 0.01, ***p ≤ 0.001, ****p ≤ 0.0001).

To examine if reduced NET formation is truly a consequence of 4-OI treatment, basal parameters of neutrophils were evaluated (**Figure 2**). To stay viable *ex vivo*, neutrophils must adhere. As we shown in **Figure 2A**, the adherence ability of neutrophils (CV assay) after treatment with 4-OI was affected only by its higher concentration (**Figure 2A**) whereas viability (PrestoBlue) was not altered (**Figure 2B**). To make sure the detected differences in cell adherence/numbers did not influence data shown in **Figure 1** on the capacity of neutrophils to cast NETs, we recalculated NET release to the numbers of adherent cells but we still detected weaker NET release in the presence of 4-OI (**Supplementary Figure 4**). Importantly, we detected that 4-OI itself increases the metabolic activity (MTT assay) of neutrophils at all tested concentrations but this parameter was not altered once the cells were subsequently stimulated with LPS (**Figure 2C**). Additionally, we did not observe differences in the capacity of neutrophils to release ROS (**Figure 2D**). DMSO solvent in which 4-OI was resuspended (stock) did not affect any of the tested parameters.

### Formation of NETs by Lean and Obese Mouse-Derived Neutrophils Is Diminished by Itaconate

**Figure 3:**
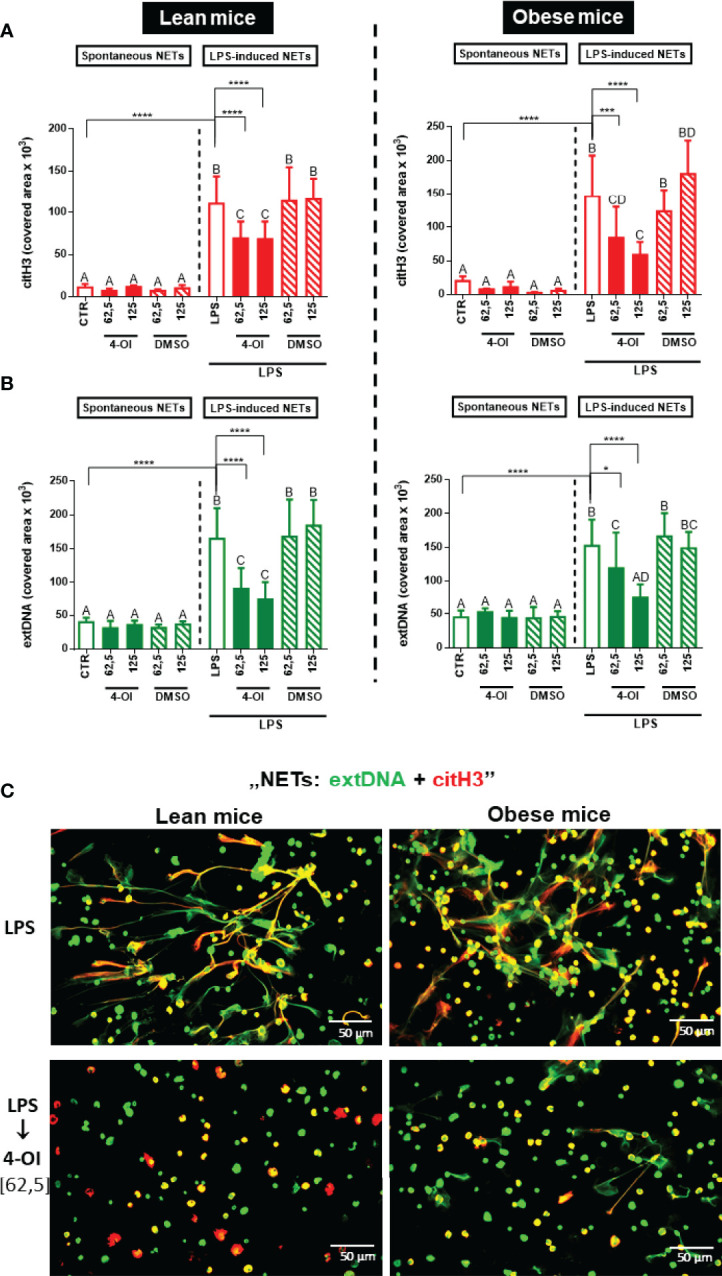
Quantification of neutrophil extracellular trap (NET) formation by neutrophils of lean and obese mice upon 4-octyl itaconate (4-OI) treatment. Neutrophils isolated from the bone marrow of lean (control diet) and obese (high fat diet) mice were treated with 4-OI at concentrations of 62,5 µM and 125 µM for 1 hour. The remaining control groups of cells were treated with dimethylsulfoxide (DMSO) solvent in the same volume as it was used in 4-OI working solution (62,5 µM; 125 µM) or they were left unstimulated (CTR). Some of the cells were subsequently stimulated for 6 hours with lipopolysaccharide (LPS) at a concentration of 75 μg/ml. Quantification of NET formation: **(A)** area covered by the citrullinated histone H3 (citH3) signal and **(B)** area covered by extracellular DNA (extDNA) signal (left panel – lean mice, right panel – obese mice). **(C)** Representative images of neutrophils isolated from lean (left panel) and obese mice (right panel): NETs were visualized by co-staining of citrullinated histone H3 (citH3, red) and extDNA (green). The results are expressed as the mean values ± SD; n≥3. Values significantly different between the groups (p < 0.05) according to one-way ANOVA (*post hoc* Bonferroni test) are designated by letters, where the same letter indicates no differences between groups (different letters indicate statistical differences). Additionally asterisks indicate significant differences between groups according to unpaired two-tailed Student’s t-test (*p ≤ 0.05, ***p ≤ 0.001, ****p ≤ 0.0001).

In order to verify if the impaired NET formation after 4-OI treatment depends on the metabolic state of animals, we tested also neutrophils collected from lean and obese mice. In line with our previous findings (13), neutrophils isolated from obese mice formed more NETs than those isolated from lean animals (**Supplementary Figures 5A, B**). Importantly, however, pretreatment of cells isolated from lean/obese mice with 4-OI highly decreased NET release (extDNA and citH3) independently of the metabolic state of the host mice (**Figures 3A–C**) and in a similar pattern as observed in **Figure 1**. Additionally, we examined basal parameters of neutrophils isolated from lean and obese mice, and as anticipated, in the studied individuals itaconic acid did not affect the adherence (**Supplementary Figure 6A**), viability (**Supplementary Figure 6B**), metabolic activity (**Supplementary Figure 6C**), and ROS production (**Supplementary Figure 6D**) by LPS stimulated cells.

### Itaconate Inhibits NET Formation Independently of the Age of Mice

Another factor that can influence neutrophil capacity to release NETs is the biological age of individuals (both mice and humans) from which they are isolated. Therefore we also compared the capacity to cast NETs by neutrophils collected from young (5-6 weeks old; Young, **Figure 1**) individuals with those of 6 months old mice (Old, **Figure 3** – lean mice; the comparison in **Supplementary Figure 7A, B**). Also in this case, we were able to observe the inhibitory effect of 4-OI on NET formation although neutrophils of old mice were releasing much less NETs than those coming from their younger counterparts (**Supplementary Figures 7A, B**).

### Itaconic Acid Increases Nrf2 and HO-1 Activity but Decreases Hif-1α in Relation to NET Release

**Figure 4:**
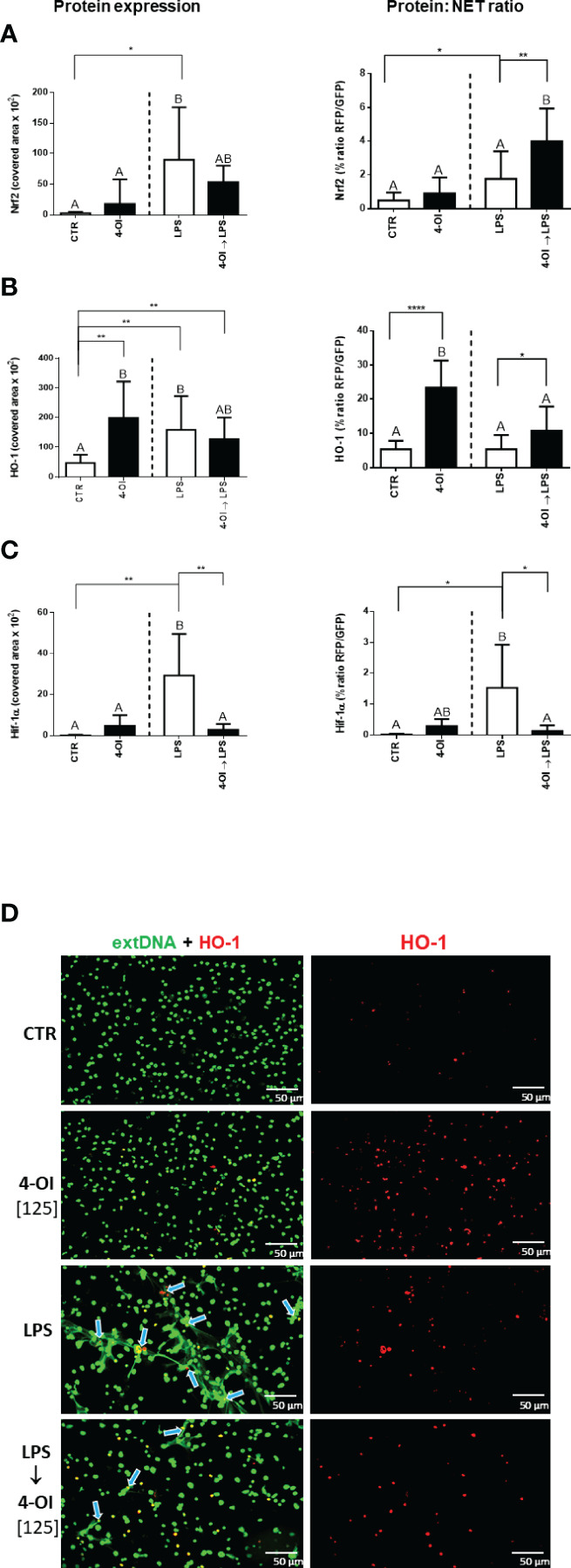
Expression of nuclear factor erythroid 2-related factor 2 (Nrf2), heme oxygenase (HO-1) and hypoxia-inducible factor-1α (Hif-1α) in relation to NET release by neutrophils treated with 4-octyl itaconate (4-OI). Neutrophils isolated from healthy mice on a standard diet were either pre-treated with 4-OI at a concentration of 125 µM (for 1h; 4-OI), incubated with lipopolysaccharide (LPS) at a concentration of 75 μg/ml (for 6h), pre-treated with 4-OI and stimulated with LPS (4-OI→LPS) or left unstimulated (CTR). Quantification of Nrf2 **(A)**, HO-1 **(B)** and Hif-1α **(C)** expression: left panel – basic expression, right panel - ratio of the given protein to NETs (the extDNA positive area). Representative images of NETs formed upon the above treatments **(D)**. Extracellular DNA (extDNA) is shown in green and HO-1 is shown in red. To visualize co-localization of extDNA with HO-1 expression, the images from each channel were overlaid. NETs – HO-1 co-location is marked with blue arrows. The results are expressed as the mean values ± SD; n≥3. Values significantly different between the groups (p < 0.05) according to one-way ANOVA (*post hoc* Bonferroni test) are designated by letters, where the same letter indicates no differences between groups (different letters indicate statistical differences). Additionally asterisks indicate significant differences between groups according to unpaired two-tailed Student’s t-test (*p ≤ 0.05, **p ≤ 0.01, ****p ≤ 0.0001).

To study the mechanism of the inhibition of NET formation by itaconic acid, we verified expression of Nrf2, HO-1 and Hif-1α because their altered (increased/decreased) expression/activity had been previously described in macrophages treated with 4-OI (26, 31). We presented the results of this experiment in two forms: expression of Nrf2, HO-1 and Hif-1α as it was detected itself (left panel) and expression recalculated against the amount of formed NETs (right panel; expression: NETs ratio) because we observed that the expression of proteins occurs mostly in places co-localizing with NETs. We report that LPS itself increased Nrf2 expression whereas additional pretreatment with 4-OI downregulated it although there was still a tendency to its higher expression than in CTR and 4-OI alone groups (**Figure 4A** left panel; **Supplementary Figure 8**). When Nrf2 levels were correlated to the release of NETs (Nrf2:NET ratio; **Figure 4A** right panel), increased Nrf2 levels were detected only when neutrophils were pre-treated with 4-OI followed by LPS stimulation (**Figure 4A**, right panel). When basic expression of HO-1 was evaluated it was increased by 4-OI, LPS and a combination of the two (**Figure 4B**, left panel; **Figure 4D**). Interestingly, although ratio of HO-1 to NETs was still strongly enhanced by 4-OI itself and to some extent by 4-OI→LPS combination, LPS alone did not affect HO-1:NET expression (**Figure 4B**, right panel). Finally, 4-OI significantly decreased expression of Hif-1α which was otherwise elevated by LPS (**Figure 4C**). The pattern of Hif-1α expression was the same when recalculated to numbers of NETs (**Figure 4C** left vs. right panel).

### Inhibition of Hif-1α Diminishes the Formation of NETs

**Figure 5:**
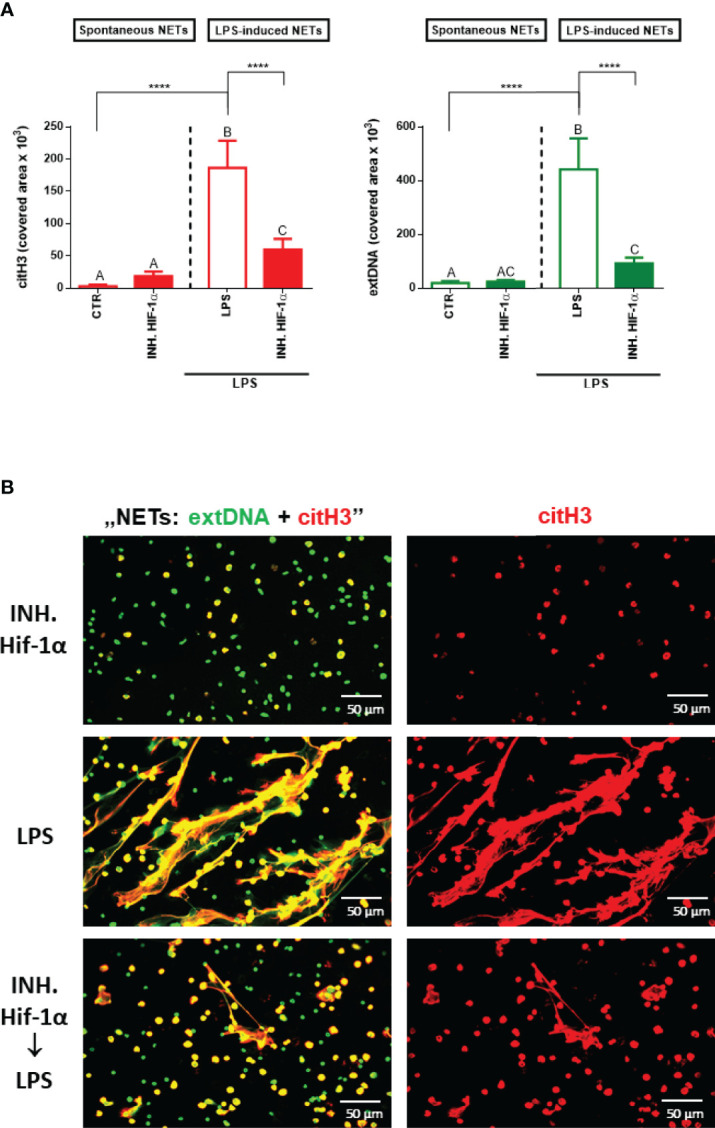
Effects of the Hif-1α inhibition on the ability to form NETs by neutrophils isolated from healthy mice on a standard diet. Neutrophils isolated from the bone marrow of mice on a standard diet were treated with Hif-1α inhibitor at a concentration of 10 µM for 1 hour. Some cells were subsequently stimulated for 6 hours with lipopolysaccharide (LPS) at a concentration of 75 μg/ml. The control group consisted of cells untreated with Hif-1α and unstimulated with LPS (CTR). Quantification of NET formation **(A)**: area of citH3 and extDNA signal. **(B)** Representative images of NETs formed upon the above treatments. The results are expressed as the mean values ± SD; n≥3. Values significantly different between the groups (p < 0.05) according to one-way ANOVA (*post hoc* Bonferroni test) are designated by letters, where the same letter indicates no differences between groups (different letters indicate statistical differences). Additionally asterisks indicates significant differences between groups according to unpaired two-tailed Student’s t-test (****p ≤ 0.0001).

To validate our hypothesis that itaconic acid inhibits NET release through the Nrf2/HO-1/Hif-1α-dependent pathways, firstly we examined the effect of a Hif-1α inhibitor on the ability of neutrophils to form NETs. Indeed, inhibition of Hif-1α ceased NET release that was induced by LPS (**Figure 5A, B**). Itself, the Hif-1α inhibitor did not alter NET formation.

### HO-1 Is Involved in the Impact of Itaconate on NET Release

**Figure 6:**
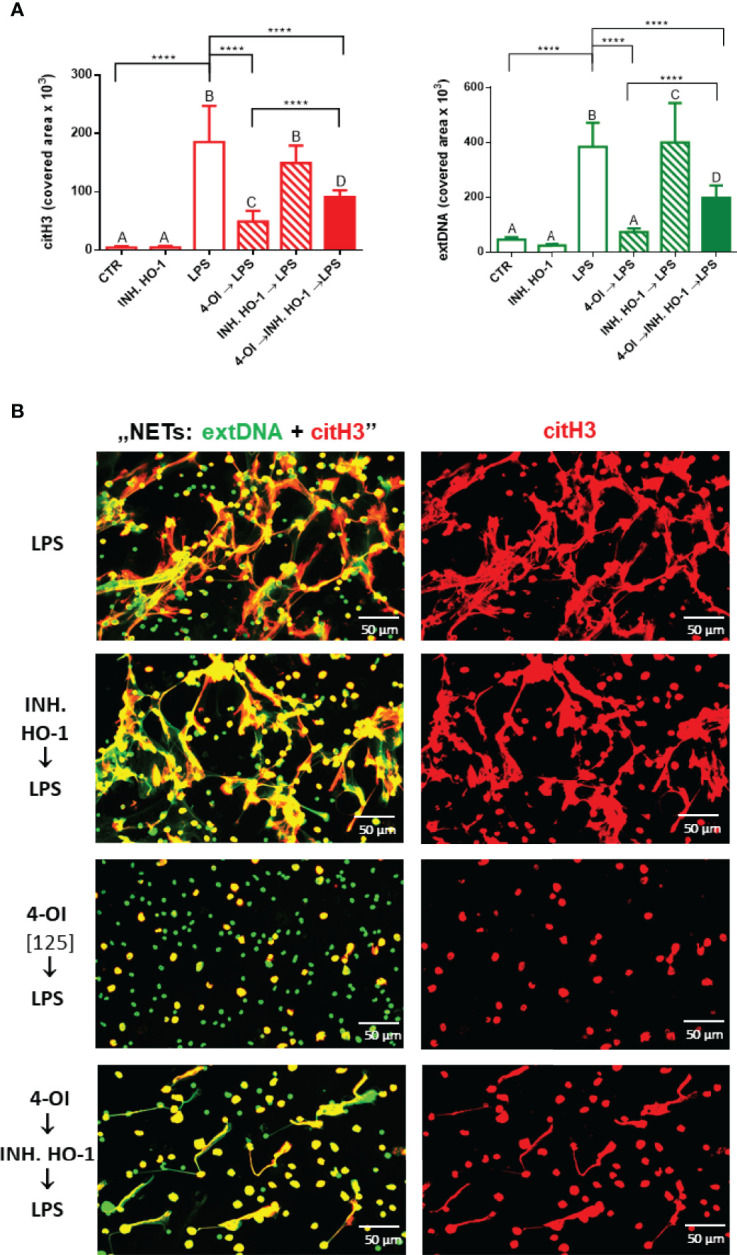
Effects of HO-1 inhibition on the ability to form NETs by neutrophils isolated from healthy mice on a standard diet. The following regimes of neutrophils isolated from healthy mice on a standard diet were tested: stimulation with lipopolysaccharide (LPS) at a concentration of 75 μg/ml; pretreatment with 4-OI at a concentration of 125 µM (4-OI) followed after 1 hour by stimulation with LPS (4-OI→LPS); pretreatment with HO-1 inhibitor (Tin Protoporphyrin IX dichloride, 20 µM) and subsequent stimulation with LPS after another hour (INH.HO-1→LPS); pretreatment with 4-OI followed after 1 hour with HO-1 inhibitor (Tin Protoporphyrin IX dichloride, 20 µM) and subsequent stimulation with LPS after another hour (4-OI→INH.HO-1→LPS). Some cells were left untreated (CTR). Quantification of NET formation **(A)**: area of citH3 and extDNA signal. **(B)** Representative images of NETs formed upon the above treatments. The results are expressed as the mean values ± SD; n≥3. Values significantly different between the groups (p < 0.05) according to one-way ANOVA (*post hoc* Bonferroni test) are designated by letters, where the same letter indicates no differences between groups (different letters indicate statistical differences). Additionally asterisks indicate significant differences between groups according to unpaired two-tailed Student’s t-test (****p ≤ 0.0001).

As data presented in **Figure 4B** (right panel) indicated, itaconate itself dramatically increased expression of HO-1 relative to NETs whereas LPS alone did not enhance it if the protein expression is recalculated against the amount of NETs. Therefore it was not surprising that the HO-1 inhibitor added to neutrophils prior to LPS did not alter NET release (**Figures 6A, B**). To induce HO-1 expression we pre-treated the cells with 4-OI and after 1 hour we added the HO-1 inhibitor to counteract action of 4-OI in terms of HO-1 expression, but without blocking other possible pathways. We observed partially diminished NET formation upon 4-OI/HO-1 inhibitor/LPS, i.e. more NETs were released than upon 4-OI/LPS treatment but significantly less in comparison to neutrophils stimulated with LPS only (**Figures 6A, B**). This result suggests that also other factors contribute to the effect of 4-OI as inhibitor.

## Discussion

NET formation is beneficial at the early stages of infection due to their ability to trap or even kill immobilized pathogens (32), but the process is detrimental during sterile inflammation or later on during the inflammatory response initiated by infection (8). This is because NET components persist in the vasculature and cause collateral damage. Therefore it is of high importance to establish means by which NET formation could be controlled. Currently, no specific inhibitors exist and to constraint the process either inhibitors of citrullination are used such as Cl-amidine or blockers of reactive oxygen species (ROS) formation or NE activity inhibitors. During citrullination driven by peptidylarginine deiminase 4 (PAD4) a charge of amino acids changes to neutral leading to destabilization of chromatin and its decondensation allowing for NET release (33); also ROS and NE are required for the initiation and/or execution of NET formation (7, 34, 35). However, there are also PAD4- (36), ROS- (37) and NE- independent (38) paths of NET release which limit application of the respective inhibitors. However, recently new classes of NET inhibitors were also reported such as lipid carriers (ROS-dependent NETs only (39) or tetrahydroisoquinolines (40). The latter examples also show that organic substances, even of exogenous origin, are good candidates for NET modulation. Therefore we turned our attention to endogenously produced itaconate which is a product of interrupted Krebs cycle in macrophages (22). Of importance, changes in the Krebs cycle that lead to itaconate formation are related to inflammatory stimuli, namely LPS action on macrophages (26).

Itaconate was shown to display anti-inflammatory functions in multiple settings and models. For example, itaconate pretreatment of LPS-stimulated murine bone marrow-derived macrophages (BMDMs) prevented activation of M1 macrophages *in vitro* (it led to the inhibition of inflammasome, IL-1β/IL-6/IL-12p70) in a Irg1-dependent manner (41) Immune-responsive gene 1 (Irg1) is essential for itaconate synthesis in macrophages (41). Importantly, itaconate did not block all cytokine production as TNF-α was unaffected by it, thus excluding a possibility that the metabolite halted total NF-κB signaling (42). Importantly, the anti-inflammatory action of itaconate was also shown *in vivo*, e.g. in a model of *Mycobacterium tuberculosis* infection (43, 44). Unlike wild-type mice, *Irg*1^−/−^ animals did not survive it due to an increased infection and excessive inflammation caused by neutrophils. Knockout of Irg1 expression in myeloid cells other than neutrophils lead to the above phenotype whereas Irg1 in neutrophils had little effect on the inflammation (in detail: cytokine release). This shows that *in vivo* regulation of neutrophil activity is under Irg1 control expressed in other cells (44) and thus strengthens our hypothesis that Irg1-dependent itaconate might modulate neutrophil activity. Herein we concentrated on the release of neutrophil extracellular traps due to their detrimental role during multiple inflammatory or metabolic disorders (8). We observed a significant inhibition of NET release in the presence of either concentration of 4-OI tested by analyzing either citrullinated histone 3 (citH3) or extracellular DNA (extDNA) in images and quantitatively. Importantly, we observed the same effect in neutrophils isolated from obese mice and independently of animal’s age. Previously we reported that neutrophils of obese mice cultured *ex vivo* form NETs in a similar manner as those of lean animals (13). Furthermore that cells isolated from mice with ongoing sepsis release even higher quantities of traps without stimulation (so called spontaneous NETs) (17). However, we observed differences in metabolic pathways involved with an evident engagement of glycolysis and PPP in the case of neutrophils collected from lean mice. However, in obese individuals these pathways were utilized only to release NETs spontaneously while after LPS stimulation they exhibited so called “exhausted phenotype” with diminished NETs formation despite high glycolytic potential and flexibility to oxidize fatty acids (17). As we report here, despite these differences in the engagement of various metabolic pathways, itaconate acted on neutrophils of either lean or obese origin in the same suppressive manner. Furthermore, it is also established that capacity to cast NETs undergoes regulation by immunoaging. At the cellular level, immature neutrophils cast less or no NETs when stimulated but tend to spontaneously release NETs, whereas senescent neutrophils form more NETs upon stimulation (45). But also age of an individual shapes neutrophil capacity to cast the traps. The immune system matures during development and then declines as we age. However, NET formation in human infants/neonates is weaker than in children and adults due to endogenous inhibitors of NETs (46), and the same pattern is observed in elderly individuals (45, 47). Immunosenescence is associated with low-grade chronic inflammation resulting from an imbalance between pro- and anti-inflammatory factors (48), however, upon insult the immune response is usually weaker. Despite differences in neutrophil activity and NET casting in regard to an individual age, we still detected the impact of itaconate on neutrophils collected from older mice (lean controls for the obese mice; due to the time required for fattening both groups of mice were app. 24 weeks old at the time of experiments; **Figure 3**) in comparison to animals app. 8 weeks old that were used in the majority of experiments (**Figures 1**, **2**, **4**–**6**; comparison of young vs. old – **Supplementary Figure 7**). In these mice NET formation was decreased by itaconate although indeed NET numbers were significantly smaller than in younger animals. Interestingly, likewise obesity is accompanied by low-grade chronic inflammation and also in obesity, as in older individuals, increased quantities of soluble NE are detected (45, 49). When we compared NET formation by neutrophils of obese and lean animals we observed the same trend of more traps being released by the former and even upon LPS stimulation. Still, itaconate was inhibiting NET formation in either group. Therefore itaconate did inhibit trap release independent whether the process was diminished in a given neutrophil population (the cells from older mice) or increased (the obese animals).

In macrophages, itaconate acts by inhibiting succinate dehydrogenase (SDH) in mitochondria. This is in line with a fact that mitochondria play a pivotal role in macrophage response to bacterial pathogens but also regulate energy metabolism, lipid synthesis, and autophagy in this cell type (41). However, when we performed the MTT assay we did not observe a significant inhibition of the response (there was even an transient increase of the signal due to the DMSO solvent). The tetrazolium salt MTT is reduced to water insoluble purple formazan crystals in the metabolically active cells by mitochondrial dehydrogenases, predominantly, but not exclusively, SDH (50). Since neutrophils, unlike macrophages carry very few mitochondria (15), and SDH is the major enzyme metabolizing MTT, we hypothesized that in neutrophils itaconate acts differently. This seems to be further confirmed by a lack of impact of itaconate on the generation of ROS by neutrophils whereas in the case of macrophages, SDH inhibition can reduce the production of ROS derived from succinate oxidation by SDH (51). Nevertheless, even in the case of macrophages itaconate is considered to be a comparatively weak inhibitor of SDH (26). In line with this subsequent studies enlightened other mechanisms of its anti-inflammatory action. Namely, itaconate was shown to activate Nrf2 and in this way inhibit IL-1β synthesis by macrophages. As a consequence of itaconate-driven post-translational modification, Nrf2 is liberated and translocates to the nucleus where it initiates transcription of antioxidant genes such as those encoding NAD(P)H dehydrogenase 1 [quinone] (NQO1), glutathione (GSH) and HO-1 (26). NQO1 stabilizes p53, protecting it from degradation, and GSH neutralizes ROS. HO-1 is primarily considered in the context of heme degradation upon release of hemoglobin from hemolyzed erythrocytes as it occurs in some types of anemia or during sepsis but it also functions as a ROS scavenger (20). Although no direct studies on a connection between HO-1 and NET formation were performed, heme (52) and hemin (53) were shown to induce NETs one would expect HO-1 to counteract this process. Especially given that HO-1 deficiency promotes the severity of sepsis (54), HO-1 in neutrophils attenuates their infiltration during sepsis (55), and it down-regulates NADPH oxidase subunits (thus ROS synthesis) in inflammatory neutrophils (56). However, upon application of an HO-1 inhibitor prior to LPS we did not detect any significant impact on NET release indicating that HO-1 itself is not involved in this process. Interestingly however, we show an increase in the expression of HO-1 in neutrophils pre-treated with 4-OI prior to stimulation with LPS and especially strong upregulation of HO-1 by itaconate itself. When we subsequently abolished HO-1 expression induced by 4-OI we observed a partial reversal of NET inhibition indicating that HO-1 is to some extent involved in the effects of itaconate in regard to the trap casting downregulation. However, it seems that HO-1 is not critical for the process (statistically significant but only limited effect) and thus possibly acts *via* control of other molecules that might be altered by 4-OI. The latter conclusion is strengthen by a fact that although HO-1 expression is increased by LPS alone (the levels of the protein itself), once its expression is recalculated to NETs (the ratio) it is weak as in control unstimulated cells, and inhibition of HO-1 prior to LPS alone does not impact NETs. Thus we speculated that 4-OI might impact also other molecules which are under control of HO-1 or control HO-1 such as hypoxia-inducible factor 1α (Hif-1α). HO-1 can stabilize Hif-1α e.g. during ischemia (29), and in turn Hif-1α can mediate transcriptional activation of the HO-1 gene in response to hypoxia (57, 58). Hif-1α upregulates genes promoting cell survival such as glycolytic enzymes and its inhibition/deletion in macrophages leads to dramatically decreased ATP levels and subsequent impairment in their motility and bacterial killing (30). Accordingly, Hif-1α knockout mice are protected from LPS induced mortality (30). In neutrophils, an increase in Hif-1α causes a metabolic shift toward glycolysis which mainly fuels these cells in ATP. In line with this and previous findings on glycolysis requirement for NET release (16, 17), in our current studies Hif-1α expression correlated with NET release upon LPS, and inhibition of Hif-1α completely prevented NET formation. In agreement with the above, McInturff et al. (59) demonstrated upregulation of Hif-1α during NET formation following LPS stimulation in primary human neutrophils in an mammalian target of rapamycin (mTOR)-dependent manner (59).

**Figure 7:**
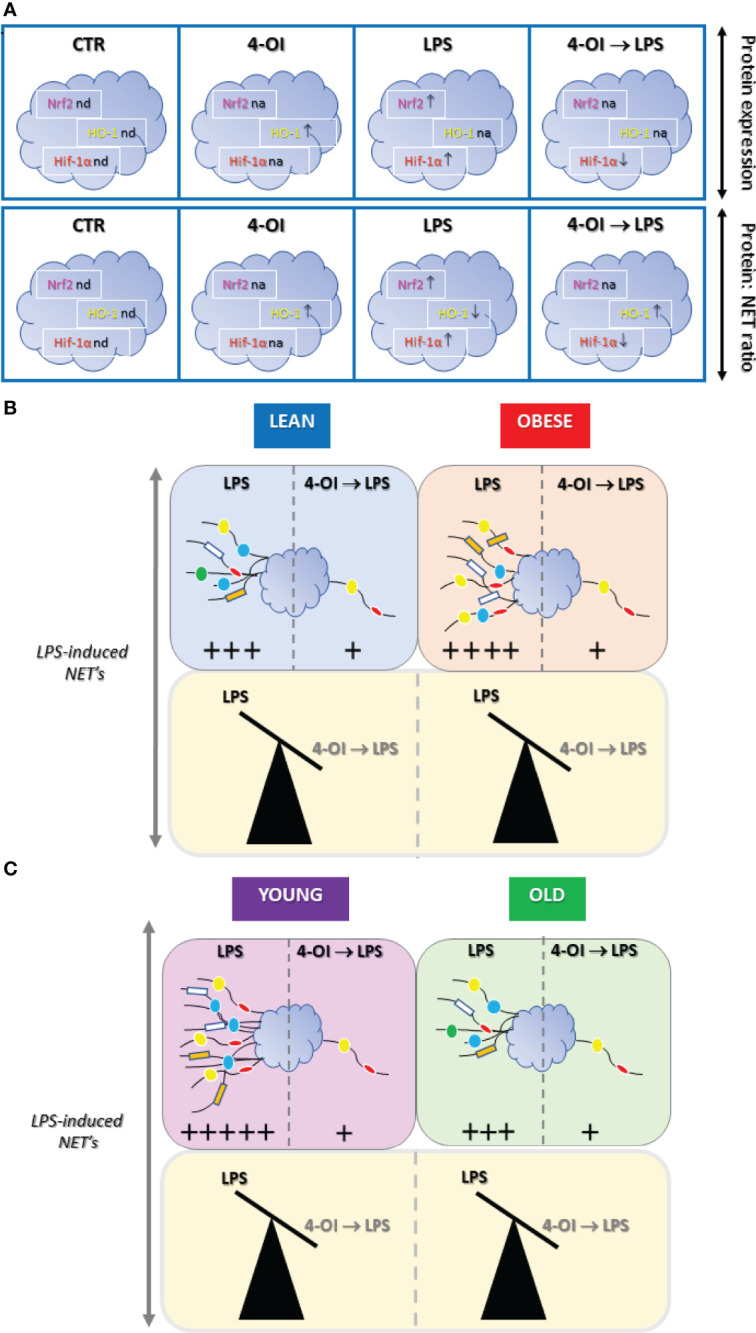
Summary of main results on the effect of itaconate (4-octyl itaconate, 4-OI) on NET release and expression of Nrf2, HO-1 and Hif-1α by murine neutrophils stimulated with lipopolysaccharide (LPS). **(A)** Impact of 4-OI expression of nuclear factor erythroid 2-related factor 2 (Nrf2; magenta), heme oxygenase (HO-1; yellow) and hypoxia-inducible factor-1α (Hif-1α, red) were marked with arrows indicating increase (↑) or decrease (↓) in the corresponding colors; nd - expression not detected, na - expression not altered. **(B)** 4-OI inhibits the ability to form NETs by neutrophils isolated from either lean (L; light blue), obese (OB; light red) or **(C)** young (Y; light violet) and old (O; light green) mice thus independently of metabolic and physiological parameters. In B and C differences are presented as balanced/unbalanced scales. The strength of the NET release is marked by arbitrary units (+) based on data presented in the **Figures 1**–**6** as well as **Supplementary Figures**.

The current findings confirm the importance and potential of itaconate as a therapeutic agent since we show its anti-inflammatory activity also towards neutrophils (**Figure 7**). Namely, itaconate capacity to negatively modulate an important long-term side effect of neutrophil activation i.e. NETs formation which can cause collateral damage e.g. during sepsis. In this regard one should also mention that itaconate has an anti-bacterial potential as it restricts bacterial growth (60) and in fact some bacteria produce enzymes degrading itaconate (51) clearly indicating its importance as a part of an endogenous defense circuit (61). Knowing that mechanisms of itaconate action involve rather uniformly Nrf2/HO-1/Hif-α pathway in both macrophages and neutrophils, now this metabolite should be tested in detail in the *in vivo* animal disease models. Especially considering that finding ways to control the formation of NETs it could be of high importance in the prevention of numerous pathologies.

## Data Availability Statement

The original contributions presented in the study are included in the article/**Supplementary Material**. Further inquiries can be directed to the corresponding author.

## Ethics Statement

The animal study was reviewed and approved by Local Ethical Committee No. II in Krakow No. 293/2017.

## Author Contributions

GB acquired data (*ex vivo* studies, immunocytochemistry, biochemical tests), performed all analyses, and participated in interpretation of data. IC co-provided study conception, acquired data (some *ex vivo* studies, immunocytochemistry) and co-participated in interpretation of data. EK provided study conception and design, participated in analyses and interpretation of data, and wrote the manuscript. All authors contributed to the article and approved the submitted version.

## Funding

The study was supported by grants from National Science Centre, Poland (NCN) No. 2018/29/B/NZ6/00713 (EK). The open-access publication of this article was funded by the programme “Excellence Initiative – Research University” at the Faculty of Biology of the Jagiellonian University in Kraków, Poland.

## Conflict of Interest

The authors declare that the research was conducted in the absence of any commercial or financial relationships that could be construed as a potential conflict of interest.

## Publisher’s Note

All claims expressed in this article are solely those of the authors and do not necessarily represent those of their affiliated organizations, or those of the publisher, the editors and the reviewers. Any product that may be evaluated in this article, or claim that may be made by its manufacturer, is not guaranteed or endorsed by the publisher.
