# Supplementary material for: Itaconate Suppresses Formation of Neutrophil Extracellular Traps (NETs): Involvement of Hypoxia-Inducible Factor 1α (Hif-1α) and Heme Oxygenase (HO-1)
Source: Front Immunol. 2022 Jun 28;13:864638. doi: 10.3389/fimmu.2022.864638 (PMC9273966; doi:10.3389/fimmu.2022.864638)
Supplement: Supplementary file 1 [file DataSheet_1.pdf]

Suppl. Fig. 1

A.

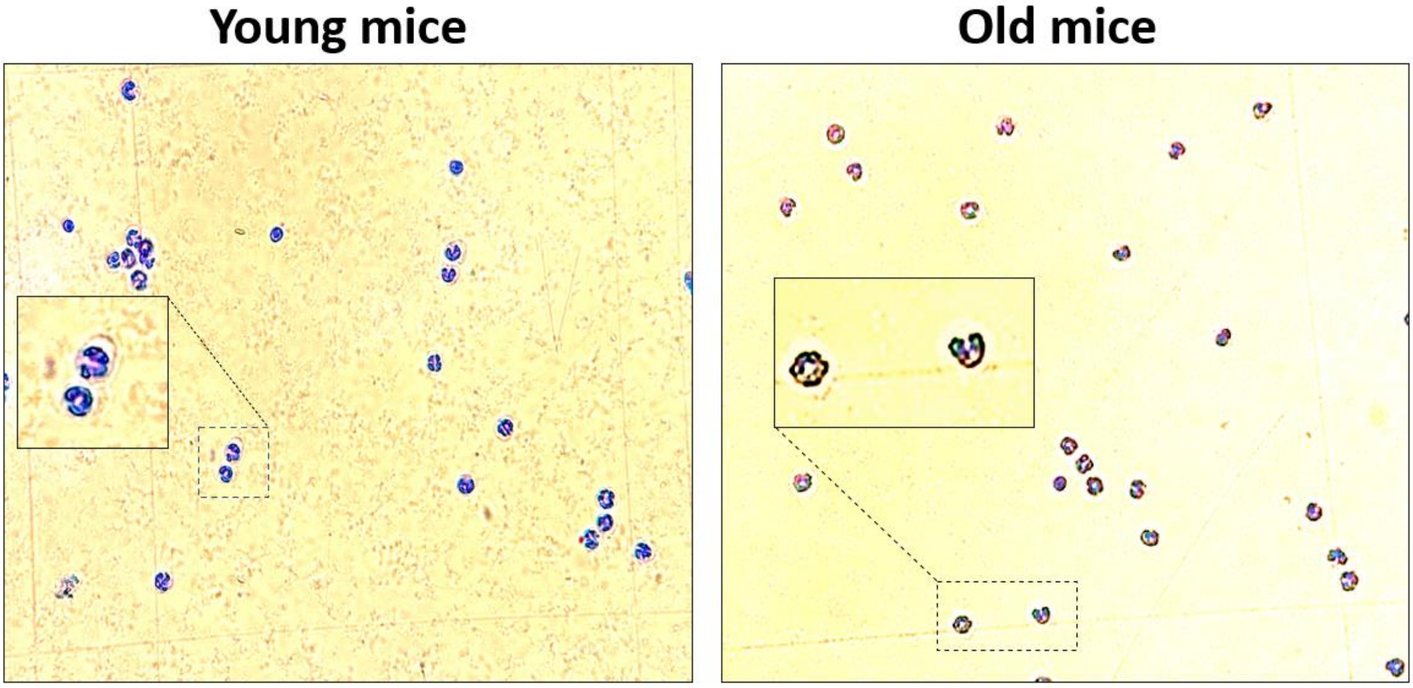

Supplementary Figure 1. Representative images taken from the Bürker chamber visualizing purity of neutrophils isolated from young and old mice. Neutrophils isolated from the bone marrow of young and old mice were stained with Türk's solution and counted in hemocytometer. Scaling – 200 x.

Suppl. Fig. 2

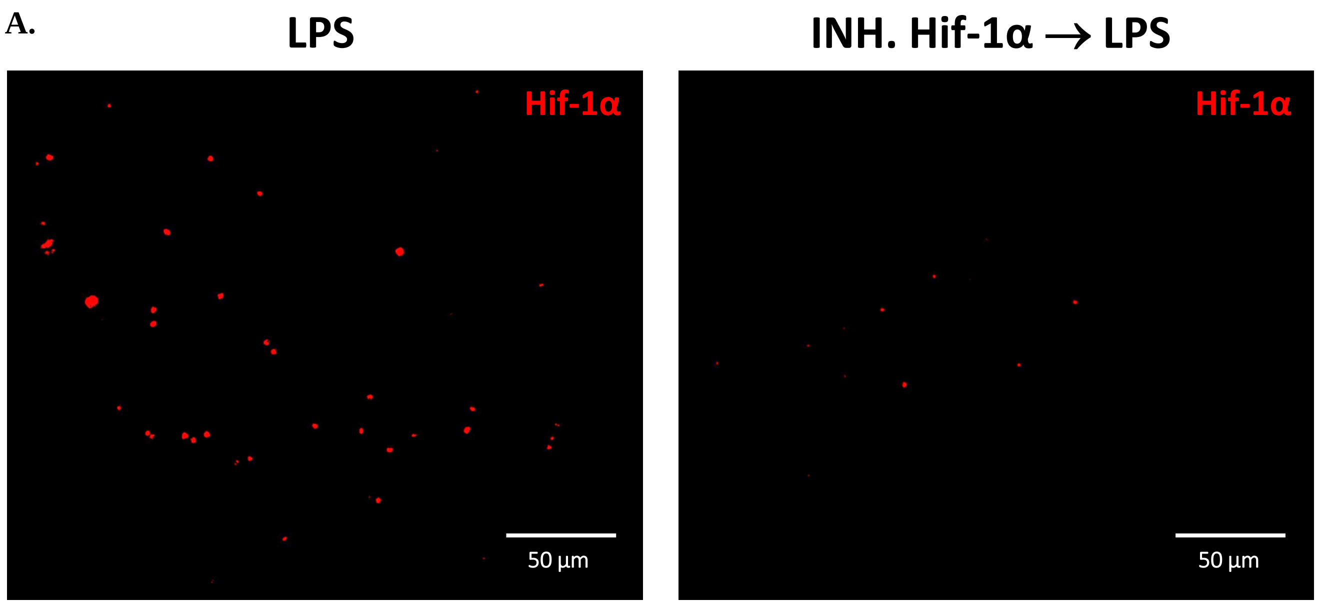

Supplementary Figure 2. Confirmation of the hypoxia-inducible factor-1α (Hif-1α) inhibitor effectivness towards Hif-1α expression blockage. Neutrophils isolated from healthy mice on a standard diet were stimulated with lipopolysaccharide (LPS) at a concentration of 75 µg/ml (for 6h), whereas some cells were at first pre-treated with the Hif-1α inhibitor at a concentration of 10 µM (for 1h; INH. Hif-1α) and then incubated with LPS. Hif-1α expression is visualized in red by immunocytochemistry.

Suppl. Fig. 3

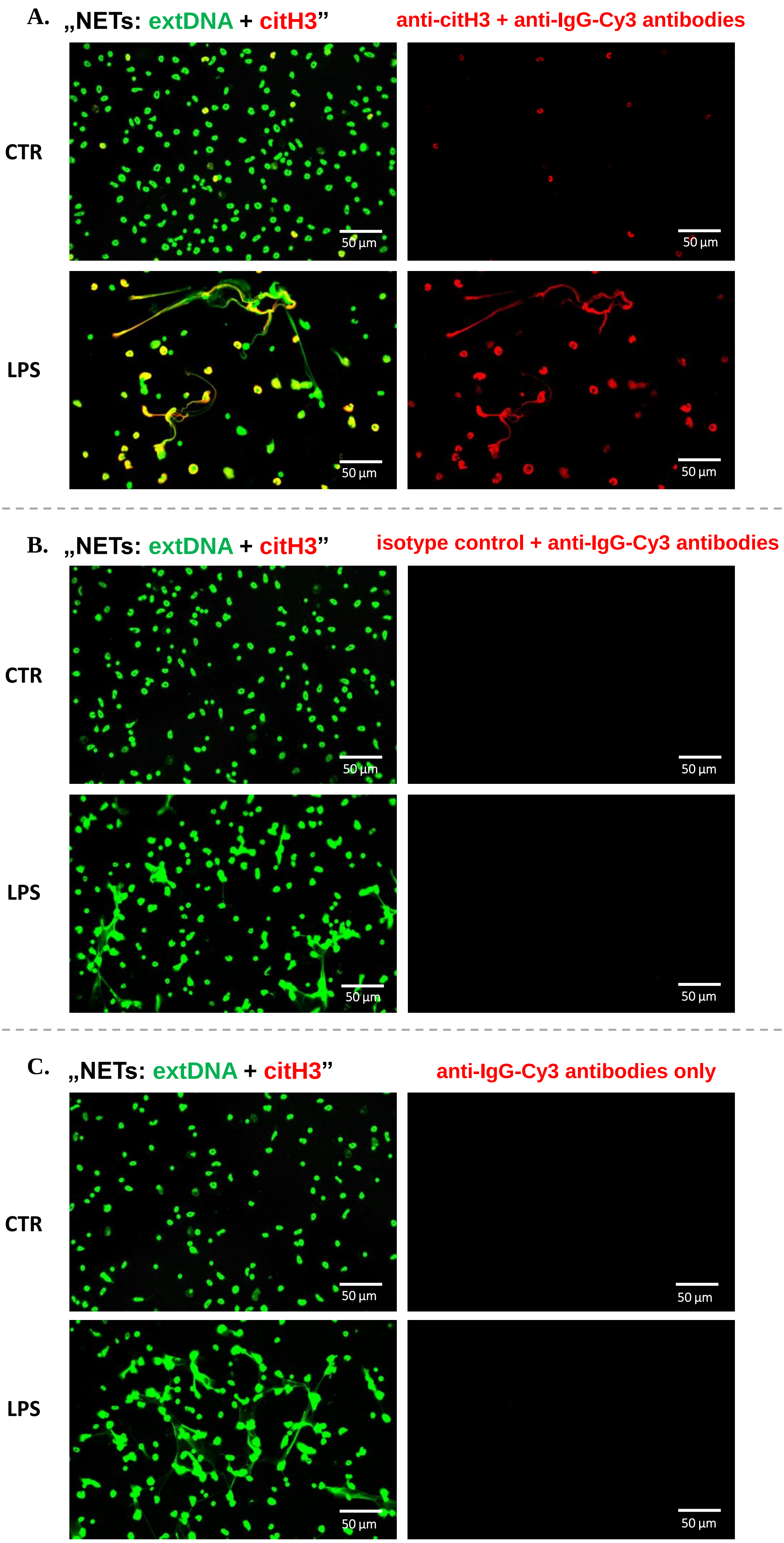

Supplementary Figure 3. Verification of specificity of the signal produced by primary anti-citH3 and secondary anti-IgG-Cy3 antibodies (immunocytochemistry). Neutrohils were either stimulated with lipopolysaccharide (LPS) at a concentration of 75 µg/ml (for 6h) or left unstimulated (CTR). Representative images show: **(A)** staining with anti-citH3 and anti-IgG-Cy3 antibodies; **(B)** the primary antibodies were replaced with the isotype-matched control antibodies, and then stained with anti-IgG-Cy3 antibodies; **(C)** the primary antibodies were omitted and anti-IgG-Cy3 antibodies were used alone. Extracellular DNA (extDNA) in green while citrullinated histone H3 (citH3) in red.

Suppl. Fig. 4

A.

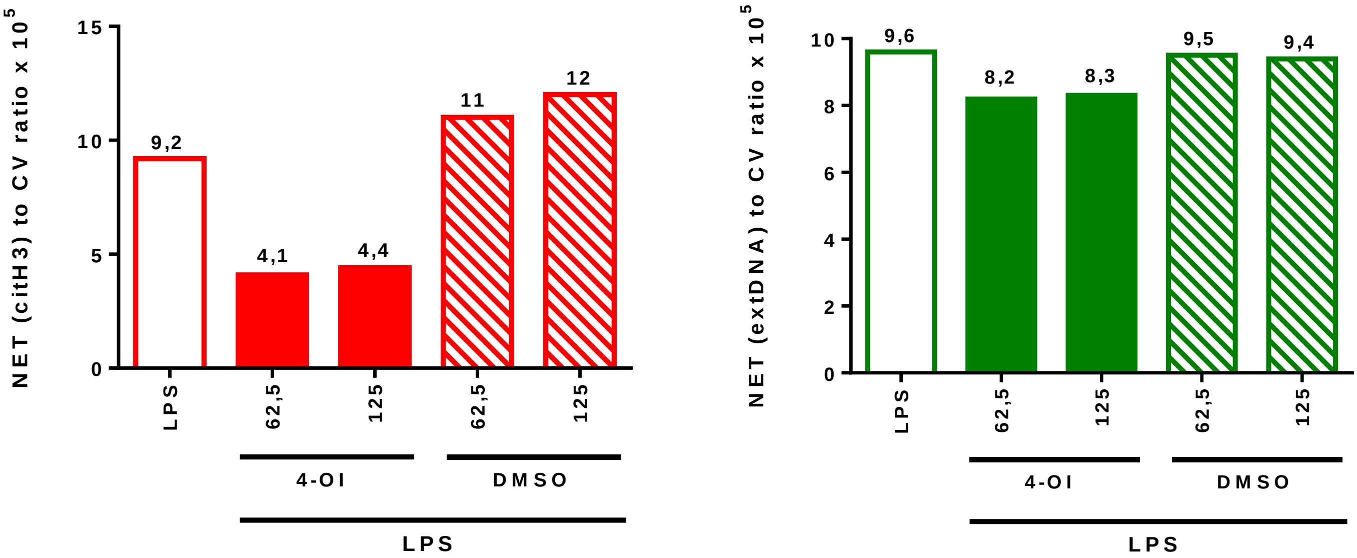

Supplementary Figure 4. Ratio of NETs released by neutrophils isolated from healthy mice on a standard diet to numbers of adherent neutrophils: results on quantification of NET release presented in Figure 1B are shown against data from Figure 2A showing data on adherent cells detected 6 hours after stimulation. Neutrophils were either pre-treated with various concentrations of 4-OI (62,5 µM; 125 µM; for 1h), incubated with lipopolysaccharide (LPS) at a concentration of 75 µg/ml (for 6h) or pre-treated with 4-OI and then stimulated with LPS. The numerical ratio is plotted above the bars.

Suppl. Fig. 5

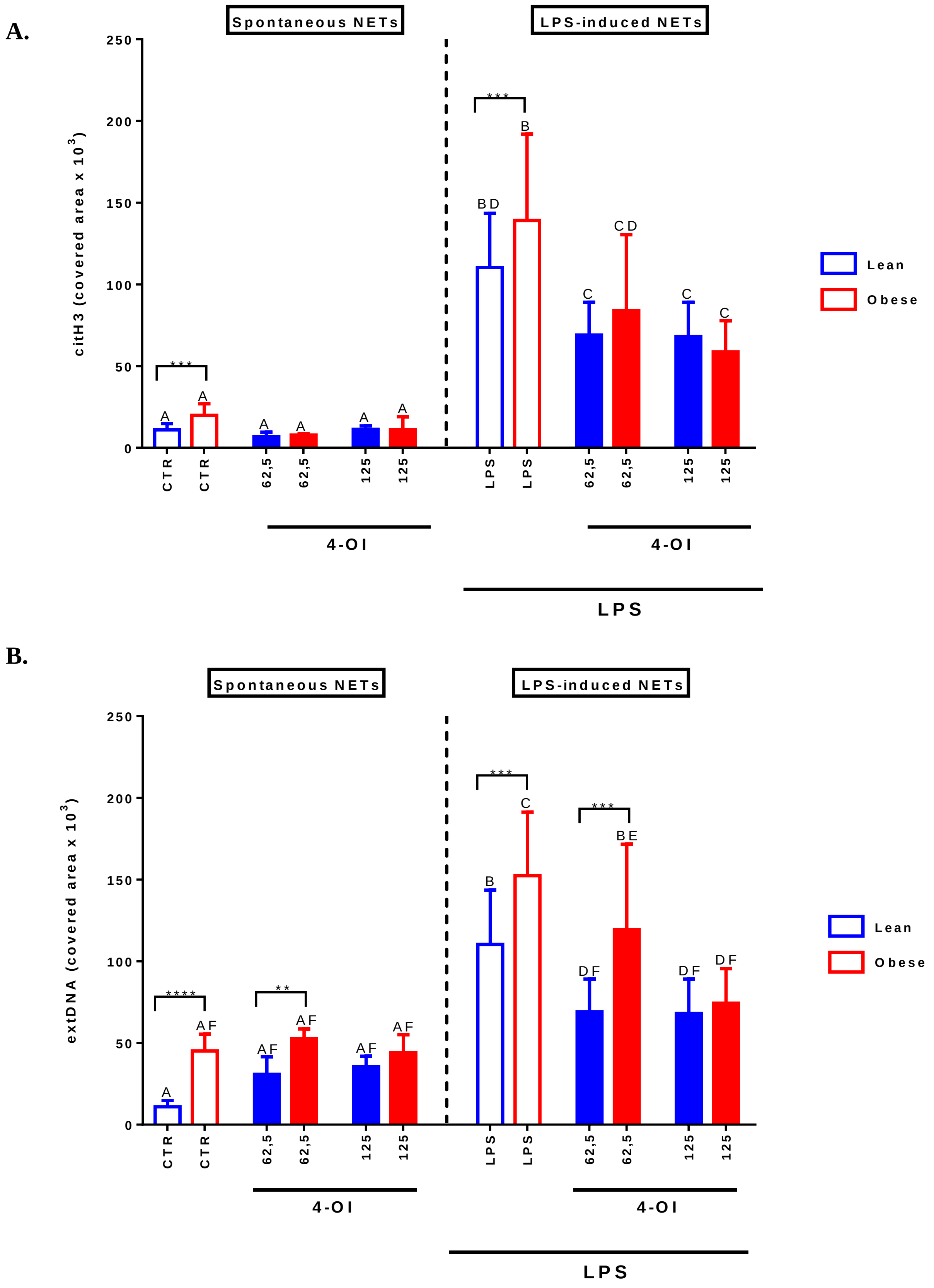

Supplementary Figure 5. Comparison of NETs released by neutrophils isolated from lean mice versus those released by neutrophils of obese mice. Statistical comparison of data from Figure 3 were results for lean and obese mice were presented separately. Neutrophils isolated from the bone marrow of lean mice on a control diet and obese mice on a high fat diet were treated with 4-octyl itaconate (4-OI) at concentrations of 62,5 µM and 125 µM for 1 hour. Some cells were stimulated for 6 hours with lipopolysaccharide (LPS) at a concentration of 75 µg/ml with or without pre-treatment with 4-OI. The control group consisted of cells untreated with 4-OI and unstimulated with LPS (CTR). Quantification of NET formation: **(A)** area of covered by citrullinated histones (citH3) and **(B)** extracellular DNA (extDNA). Values significantly different between the groups ($p<0.05$) according to one-way ANOVA (*post hoc* Bonferroni test) are designated by letters, where the same letter indicates no differences between groups (different letters indicate statistical differences). Additionally asterisks indicate significant differences between groups (lean/obese) according to unpaired two-tailed Student’s t-test (**$p \leq 0.01$, ***$p \leq 0.001$, ****$p \leq 0.0001$).

Suppl. Fig. 6

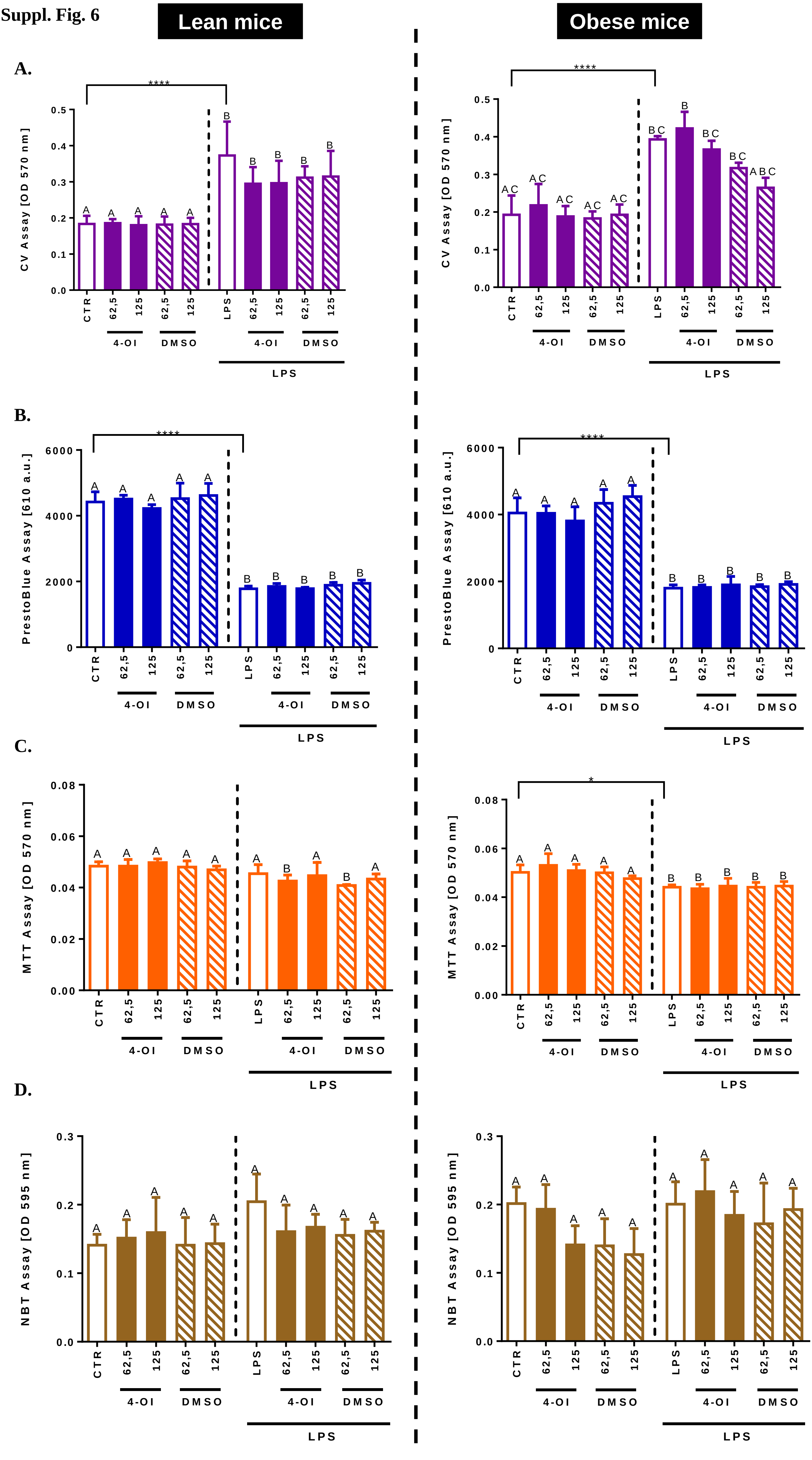
Lean mice
Obese mice
A.
B.
C.
D.
CV Assay [OD 570 nm]
PrestoBlue Assay [610 a.u.]
MTT Assay [OD 570 nm]
NBT Assay [OD 595 nm]
****
*
CTR
62,5
125
LPS
4-OI
DMSO
LPS

Suppl. Fig. 6 - continuation

Supplementary Figure 6. Effects of 4-octyl itaconate (4-OI) on the adherence to the surface, viability, metabolic activity and ROS production of neutrophils isolated from lean and obese mice. The analyses were performed with crystal violet assay (CV) **(A)**, PrestoBlue **(B)**, MTT assay **(C)** and NBT assay **(D),** respectively. Neutrophils isolated from the bone marrow of lean mice on a control diet and obese mice on a high fat diet were treated with 4-octyl itaconate at concentrations of 62.5 μM and 125 μM for 1 hour. The remaining control groups were cells treated with dimethylsulfoxide (DMSO) solvent in the same volume as was used in 4-OI working solution. Some cells were stimulated for 6 hours with lipopolysaccharide (LPS) at a concentration of 75 μg/ml. The control group was cells untreated with 4-octyl itaconate and unstimulated with LPS (CTR). The results are expressed as the mean values ± SD; $n \geq 3$. Values significantly different between the groups ($p<0.05$) according to one-way ANOVA (*post hoc* Bonferroni test) are designated by letters, where the same letter indicates no differences between groups (different letters indicate statistical differences). Additionally, asterisks indicate significant differences between groups according to unpaired two-tailed Student's t-test (*$p \leq 0.05$, ****$p \leq 0.0001$).

Suppl. Fig. 7

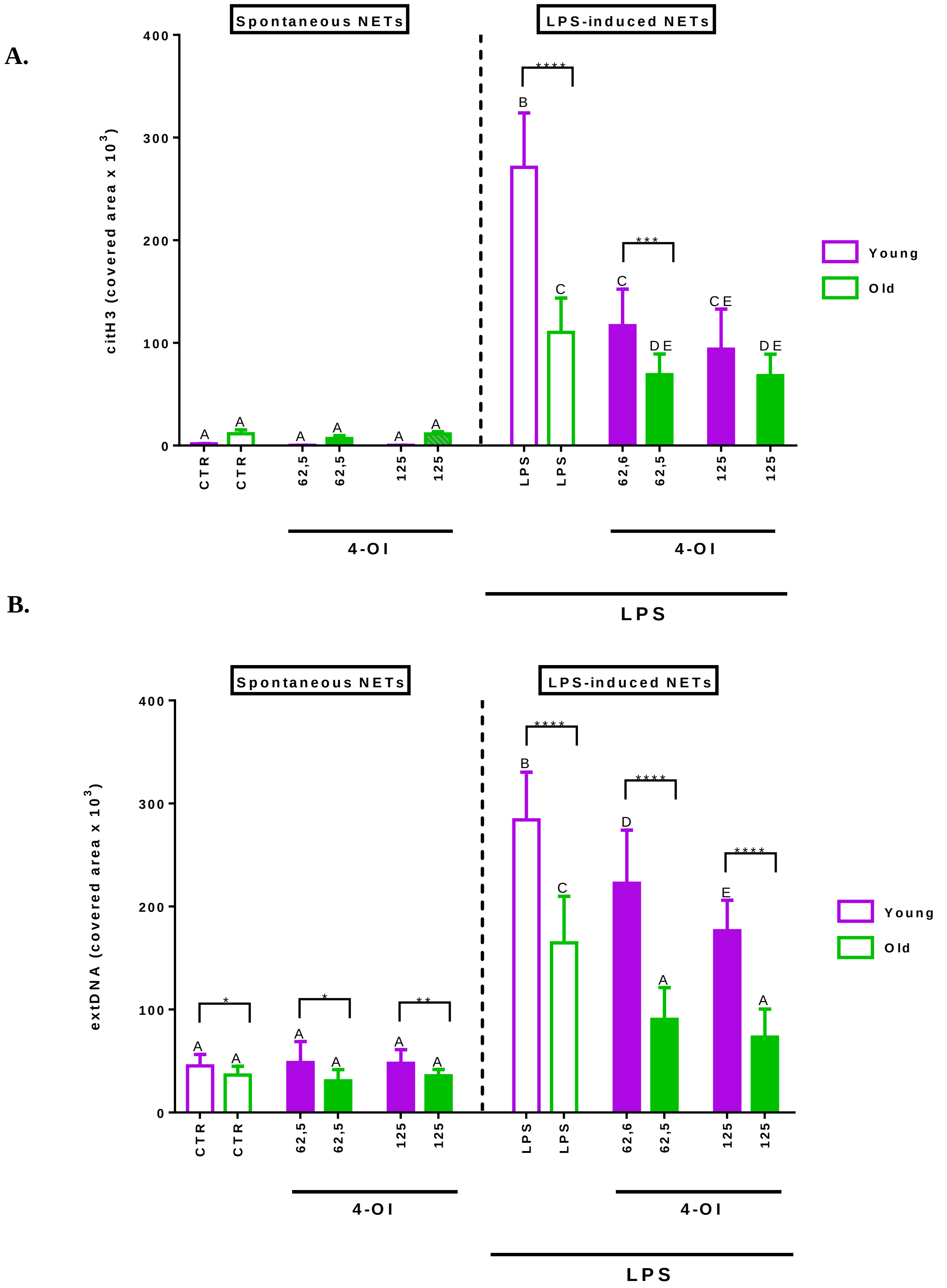

Supplementary Figure 7. Comparison of NETs released by neutrophils isolated from young mice vs NETs released from neutrophils of old mice. Statistical comparison of data from Figure 1B (young mice) and Figure 3A, B (old mice; lean controls of obese animals). Neutrophils isolated from the bone marrow were treated with 4-octyl itaconate (4-OI) at concentrations of 62,5 µM and 125 µM for 1 hour. Some cells were stimulated for 6 hours with lipopolysaccharide (LPS) at a concentration of 75 µg/ml with or without pre-treatment with 4-OI. The control group consisted of cells untreated with 4-OI and unstimulated with LPS (CTR). Quantification of NET formation: **(A)** area of covered by citrullinated histones (citH3) and **(B)** extracellular DNA (extDNA). Values significantly different between the groups ($p<0.05$) according to one-way ANOVA (*post hoc* Bonferroni test) are designated by letters, where the same letter indicates no differences between groups (different letters indicate statistical differences). Additionally asterisks indicate significant differences between groups (lean/obese) according to unpaired two-tailed Student's t-test (*p≤0.05, **p≤0.01, ***p≤0.001, ****p≤0.0001).

Suppl. Fig. 8

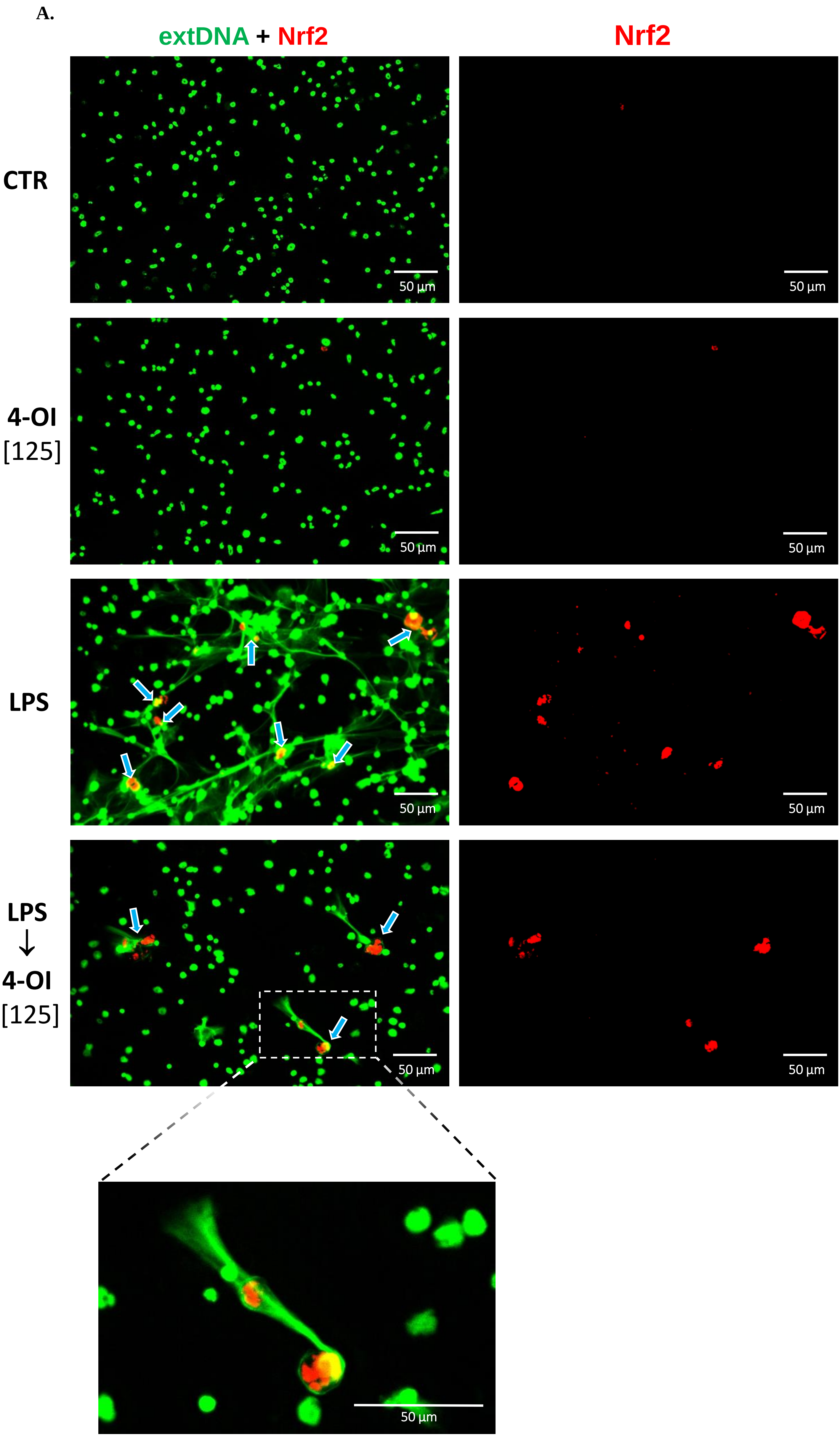

Suppl. Fig. 8 - continuation

Supplementary Figure 8. Expression of nuclear factor erythroid 2-related factor 2 (Nrf2) modulated by pre-treatment with 4-octyl itaconate (4-OI) and in relation to neutrophil extracellular trap (NET) formation. Neutrophils isolated from healthy mice on a standard diet were either pre-treated with 4-OI at a concentration of 125 μM (for 1h; 4-OI), incubated with lipopolysaccharide (LPS) at a concentration of 75 μg/ml (for 6h), pre-treated with 4-OI and then stimulated with LPS (4OI→LPS) or left unstimulated (CTR). Extracellular DNA (extDNA) is shown in green and Nrf2 is shown in red. To visualize co-localization of extDNA with Nrf2 expression, the images from each channel were overlaid. NETs - Nrf2 co-location is marked with blue arrows. Grey dashed line denotes exemplary NETs co-localizing with the Nrf2 signal (original location of the cells is marked with a white dashed line).
